# Prevalence, Pharmacological Treatment, and Control of Cardiometabolic Risk Factors among Older People in Central Stockholm: A Population-Based Study

**DOI:** 10.1371/journal.pone.0119582

**Published:** 2015-03-23

**Authors:** Rui Wang, Laura Fratiglioni, Yajun Liang, Anna-Karin Welmer, Weili Xu, Francesca Mangialasche, Kristina Johnell, Chengxuan Qiu

**Affiliations:** 1 Aging Research Center, Department of Neurobiology, Care Sciences and Society, Karolinska Institutet-Stockholm University, Stockholm, Sweden; 2 Stockholm Gerontology Research Center, Stockholm, Sweden; 3 School of Public Health, Jining Medical University, Shandong, China; 4 Karolinska University Hospital, Stockholm, Sweden; Heinrich-Heine University, Faculty of Medicine, GERMANY

## Abstract

**Background:**

Cardiometabolic risk factors and related cardiovascular diseases represent major threats to healthy aging.

**Objective:**

We aimed to estimate distribution, pharmacological treatment, and control of main cardiometabolic risk factors among older people.

**Methods:**

This population-based study included 3363 participants (age≥60 years, 64.9% women) in the Swedish National study on Aging and Care in Kungsholmen, in central Stockholm, Sweden (2001-2004). Data on demographics, cardiometabolic risk factors (hypertension, obesity, diabetes, and high cholesterol), and medication use were collected through face-to-face interviews, clinical examinations, laboratory tests, and the inpatient register. Cardiometabolic risk factors were defined following the most commonly used criteria. Prevalence was standardized using local census data.

**Results:**

The age- and sex-standardized prevalence of diabetes, obesity, high cholesterol, and hypertension was 9.5%, 12.8%, 49.7%, and 74.9%, respectively. The prevalence of hypertension and diabetes increased with age, whereas the prevalence of obesity and high cholesterol decreased with age. Forty-nine percent of older adults had two or more cardiometabolic risk factors; 9.8% had three or more. Overall, 55.5% of people with hypertension, 50.3% with diabetes, and 25.0% with high cholesterol received pharmacological treatment. Of those treated pharmacologically, 49.4%, 38.1%, and 85.5% reached therapeutic goals for hypertension (blood pressure<150/90 mmHg), diabetes (glycated haemoglobin<7%), and high cholesterol (total cholesterol<6.22 mmol/l), respectively.

**Conclusions:**

Hypertension, high cholesterol, and clustering of cardiometabolic risk factors were common among older people in Stockholm, but pharmacological treatment and control of these major factors can be improved. Appropriate management of cardiometabolic profiles among older people may help improve cardiovascular health and achieve healthy aging.

## Introduction

Cardiovascular diseases (CVDs) such as coronary heart disease and stroke, as the leading causes of death, have posed a major threat to late-life survival [1,2]. In Sweden, nearly half of people aged 65 years and over are affected by some form of cardiovascular disorders [3]. Lifestyle and cardiometabolic factors are major modifiable risk factors for CVDs. Recently, a broad range of lifestyle- and metabolic-related factors have been categorized as cardiometabolic risk factors, such as hypertension, obesity, hyperlipidaemia, glucose intolerance, sleep apnea, inflammation, and stress [4,5], although consensus has yet to be reached. In the report published in 2011 by the World Health Organization (WHO) in collaboration with the World Heart Federation and the World Stroke Organization [6], hypertension, diabetes, high cholesterol, and obesity were recognized as the four major cardiometabolic risk factors that are strongly associated with CVDs. Previous studies have suggested that optimal control of cardiometabolic risk factors could substantially increase life expectancy for middle-aged (i.e., 50 years), elderly (e.g., 60+), and even very old (e.g., 75+) people [7–9]. Cardiometabolic risk factors and related disorders also contribute to late-life cognitive dysfunction and disability in basic activities of daily living [10,11], which significantly decreases the quality of life and increases the needs and costs of long-term care and social services. In addition, evidence has emerged that an aggregation of multiple cardiometabolic risk factors is associated with a substantially increased likelihood of mobility limitation and dementia [11–13]. Thus, control of cardiometabolic risk factors has been proposed as one of the main strategies in preventing not only CVDs, but also cognitive decline and functional disability [14–16].

The American Heart Association has recommended an optimal cardiometabolic profile for adults (age ≥20 years), including a blood pressure <120/80 mmHg, fasting blood glucose <5.6 mmol/l, total cholesterol <5.18 mmol/l, and body mass index (BMI) <25 kg/m^2^, as part of ideal cardiovascular and brain health [17]. However, research has revealed that very few adults achieve these ideal cardiometabolic profiles [18]. On the contrary, the suboptimal cardiometabolic components and cardiometabolic risk factors are highly prevalent among adults, especially among older people [14,18]. For instance, data from U.S. National Health and Nutrition Examination Survey (1999–2004) suggested that the age-adjusted prevalence of suboptimal cardiometabolic conditions was 39.3% for pre-hypertension, 34.8% for borderline high cholesterol, and 35.4% for overweight among people aged 25–74 years [18], and the overall prevalence of diabetes and hypertension among people aged 55 years and older (2004–2007) was ~13% and ~50%, respectively [19]. Furthermore, previous investigations have shown that the prevalence increases with age for certain cardiometabolic risk factors (e.g., hypertension) and decreases for others (e.g., obesity) among older adults [20]. However, very few population-based studies have assessed the distribution of clustering cardiometabolic risk factors among older adults living in the community [21].

In addition, current guidelines usually set optimal goals for control in blood pressure, blood glucose, and serum cholesterol for people with hypertension, diabetes, or high cholesterol, aiming to reduce the risk of CVDs [22–24]. However, previous research has shown that the rates of achieving the targeted levels of cardiometabolic risk factors are rather low, even among adults with high cardiovascular risk [25]. Furthermore, data on pharmacological treatment of cardiometabolic risk factors among older people, especially the oldest old, in Sweden, are scarce.

In the current study, we seek to investigate the distribution, aggregation, pharmacological treatment, and control of major cardiometabolic risk factors among older Swedish adults. Specifically, we aim to (1) estimate the age- and sex-specific prevalence of cardiometabolic risk factors and suboptimal cardiometabolic conditions (i.e., prehypertension, pre-diabetes, borderline high cholesterol, and overweight); (2) explore the aggregation of cardiometabolic risk factors by age and sex; and (3) investigate pharmacological treatment and control of hypertension, diabetes, and high cholesterol.

## Materials and Methods

### Participants

The study participants were derived from the population-based Swedish National study on Aging and Care in Kungsholmen (SNAC-K). SNAC-K is an ongoing multidisciplinary study of aging and health that includes a sample of people aged 60+ years who live either at home or in institutions in the Kungsholmen district, an area of central Stockholm, Sweden. The sampling is stratified by different age cohorts and years of interval for assessment, i.e., a six-year interval for young age cohorts (60, 66, 72, and 78 years) and a three-year interval for older age cohorts (81, 84, 87, 90, 93, 96, and 99+ years). This sampling strategy is based on the fact that there are more rapid changes in health and a higher attrition rate in older than young age groups. Of the 5111 persons who were initially invited for participation, 4590 were eligible and alive to participate. Of these, 1227 refused participation, thus a total number of 3363 (73.3%) persons were examined for SNAC-K during March 2001-June 2004 [11,26].

The SNAC-K was approved by the Ethics Committee at Karolinska Institutet and by the Regional Ethical Review Board in Stockholm, Sweden. Written informed consent was obtained from all participants, and from proxies in case of cognitively impaired persons.

### Data collection

Data on demographics (e.g., age, sex, and education), medical history (e.g., diabetes), and current use of medications (e.g., antihypertensive, hypoglycemic, and hypolipidemic agents) were collected through interviews by nurses and physicians at our research centre [11,26]. Participants were asked in advance to bring a list of currently used drugs to the interview. Information on use of medications was recorded according to self-report, which was further verified by inspecting drug prescriptions and containers [27]. Medical drugs were classified according to the Anatomical Therapeutic Chemical (ATC) classification system. For those who agreed to participate but who were unable or not willing to come to our centre, home visits were conducted (n = 717). Educational level was measured by the maximum years of formal schooling and divided into elementary school, high school, and university. Height and weight were measured in light clothes with no shoes. BMI was calculated as weight (kilograms) divided by height (meters) squared. Arterial blood pressure was measured twice at a 5-min interval in a sitting position on the right arm with a sphygmomanometer, and the mean of the two readings was used in the analyses. Peripheral blood samples were taken and total cholesterol and glycated haemoglobin (HbA1c) were measured [11]. Because mono-S high performance liquid chromatography was used in the analysis of HbA1c in Sweden, the value of HbA1c was added 1.1% to make it equivalent to the international value [28]. Information on health history for all participants was also available from the inpatient register that covers all hospitalizations in Sweden since 1969, in which the criteria of the ninth and tenth revisions of the International Classification of Diseases (ICD-9 and ICD-10) were used.

### Assessments of hypertension, diabetes, high cholesterol, and obesity

Hypertension was defined as blood pressure ≥140/90 mmHg or current use of antihypertensive agents (ATC codes C02, C03, C07, C08 and C09), and pre-hypertension as blood pressure of 120–139/80–89 mmHg and no use of antihypertensive agents [22,29]. Diabetes was defined as having self-reported history of diabetes, records of diabetes in the inpatient register (IDC-9 code 250 and ICD-10 codes E10-E14), use of antidiabetic agents (ATC code A10), or HbA1c ≥6.5%; prediabetes was assessed as HbA1c of 5.7–6.4% among diabetes-free participants [30]. High cholesterol was defined as non-fasting total serum cholesterol ≥6.22 mmol/l or use of cholesterol-lowering agents (ATC code C10), and borderline high cholesterol as total cholesterol 5.18–6.21 mmol/l and no use of cholesterol-lowering agents [24,31]. We defined obesity as a BMI ≥30 kg/m^2^ and overweight as a BMI of 25–29.9 kg/m^2^ [32]. The aggregation of cardiometabolic risk factors was assessed by counting the number of the four cardiometabolic risk factors (hypertension, diabetes, high cholesterol, and obesity) that an individual concurrently possessed.

### Pharmacological treatment of hypertension, diabetes, and high cholesterol, and their control among pharmacologically treated people

Pharmacological treatment of hypertension, diabetes, and high cholesterol was defined by the self-reported current use of antihypertensive, antidiabetic, and cholesterol-lowering medications, respectively [31,33]. Of individuals who were treated pharmacologically, controlled hypertension was defined as pharmacologically treated blood pressure (I) <140/90 mmHg [33], or (II) <150/90 mmHg [22]; controlled diabetes was defined as pharmacologically treated HbA1c <7.0% [33,34], and controlled high cholesterol was defined as pharmacologically treated blood cholesterol <6.22 mmol/l [31].

### Statistical analysis

Characteristics of participants by sex were compared using chi-square test for categorical and t-test for continuous variables. Because the sampling strategy in the SNAC-K was not intended to obtain a representative sample of the local older population (oldest-olds were over sampled) [26], the overall prevalence of cardiometabolic risk factors was standardized using the age- and sex-specific census data in the Kungsholmen district. The prevalence of individual cardiometabolic risk factors and their aggregation (0, 1, 2, and ≥3 cardiometabolic risk factors) was presented by sex and age. We reported proportions of pharmacological treatment and control of hypertension, diabetes, and high cholesterol by sex and age. Stata version 12.0 for Windows (StataCorp 2011, College Station, TX: StataCorp LP) was used for all analyses.

## Results

### Demographic characteristics

The demographic characteristics of the study participants by sex are reported in Table 1. The mean age of the 3363 participants was 74.0 (SD 10.7) years, 64.9% were women, and 32.7% achieved university.

**Table 1:** Demographic characteristics of SNAC-K participants by sex.

Characteristics	Total (N = 3363)	Men (n = 1181)	Women (n = 2182)	*P* ^a^
Age (years), mean (SD)	74.0 (10.7)	71.3 (9.8)	75.4 (10.7)	<0.01
Age (years), n (%)				
60	739 (22.0)	330 (27.9)	409 (18.7)	
66	565 (16.8)	239 (20.2)	326 (14.9)	
72	478 (14.2)	189 (16.0)	289 (13.2)	
78	461 (13.7)	152 (12.9)	309 (14.2)	
81	236 (7.0)	76 (6.4)	160 (7.3)	
84	224 (6.7)	68 (5.8)	156 (7.2)	
87	174 (5.2)	42 (3.6)	132 (6.1)	
≥90	486 (14.5)	85 (7.2)	401 (18.4)	<0.01
Educational level^b^, n (%)				
Elementary	590 (17.7)	168 (14.3)	422 (19.6)	
High school	1651 (49.6)	495 (42.0)	1156 (53.7)	
University	1090 (32.7)	516 (43.7)	574 (26.7)	<0.01

^a^ *P* values were for the test of differences between men and women.

^b^There were 32 subjects with missing value.

SNAC-K, Swedish National study on Aging and Care in Kungsholmen, Stockholm, Sweden; SD, Standard deviation.

### Crude and standardized prevalence of cardiometabolic risk factors

The crude prevalence of cardiometabolic risk factors ranged from 9.5% for diabetes (men vs. women: 13.6% vs. 7.2%, *P*<0.01) to 74.9% for hypertension (73.9% vs. 75.5%, *P* = 0.30) (Table 2). The crude prevalence of unfavourable cardiometabolic conditions were 19.2% for prehypertension (men vs. women: 21.0% vs. 18.2%, *P* = 0.06), 23.3% for prediabetes (22.8% vs. 23.6%, *P* = 0.84), 39.5% for overweight (46.9% vs. 35.0%, *P*<0.01), and 34.4% for borderline high cholesterol (32.1% vs. 34.7%, *P*<0.01). The crude prevalence was slightly changed after standardization by age and sex.

**Table 2:** Crude and age- and sex-standardized prevalence (per 100 population) of cardiometabolic risk factors by sex (N = 3363).

	Total			Men			Women		
Cardiometabolic	No. of	Prevalence (95% CI)		No. of	Prevalence (95% CI)		No. of	Prevalence (95% CI)	
risk factors^a^	subjects	Crude	Standardized^b^	subjects	Crude	Standardized^b^	subjects	Crude	Standardized^b^
Hypertensive status									
Prehypertension	636	19.2 (17.9–20.5)	18.0 (17.2–18.9)	246	21.0 (18.6–23.3)	20.4 (18.9–21.9)	390	18.2 (16.6–19.9)	16.8 (15.8–17.8)
Hypertension	2496	74.9 (73.4–76.4)	76.4 (75.5–77.3)	869	73.9 (71.3–76.4)	74.7 (73.1–76.4)	1627	75.5 (73.7–77.3)	77.4 (76.3–78.6)
Diabetic status									
Prediabetes	784	23.3 (21.9–24.7)	23.7 (22.8–24.7)	269	22.8 (20.4–25.2)	22.9 (21.3–24.4)	515	23.6 (21.8–25.4)	24.9 (23.7–26.0)
Diabetes^c^	318	9.5 (8.5–10.4)	9.6 (8.9–10.2)	160	13.6 (11.6–15.5)	12.9 (11.6–14.1)	158	7.2 (6.2–8.3)	8.1 (7.3–8.8)
Obese status									
Overweight	1198	39.5 (37.8–41.2)	37.2 (36.1–38.2)	536	46.9 (44.0–49.8)	45.7 (43.9–47.6)	662	35.0 (32.9–37.2)	32.7 (31.5–34.0)
Obesity	388	12.8 (11.6–14.0)	11.7 (11.0–12.4)	154	13.5 (11.5–15.5)	13.0 (11.7–14.2)	234	12.4 (11.0–13.9)	11.1 (10.3–12.0)
Total cholesterol									
Borderline high	1066	34.4 (32.7–36.1)	34.5 (33.4–35.5)	363	32.1 (29.4–34.9)	34.0 (32.2–35.7)	683	34.7 (32.6–36.8)	34.8 (33.5–36.1)
High	1523	49.7 (47.9–51.4)	48.6 (47.5–49.7)	511	46.1 (43.2–49.0)	44.8 (42.9–46.6)	1012	51.7 (49.5–53.9)	50.8 (49.5–52.2)

^a^Numbers of subjects with missing value were 48 for prehypertension and hypertension, 244 for prediabetes (due to missing in HbA1c), 329 for overweight and obesity, and 263 for high total cholesterol

^b^The prevalence was standardized using the local age- and sex-specific census data.

^c^Among people diagnosed with diabetes, 10 subjects had type I diabetes, and 378 had type II diabetes.

CI, Confidence interval; BMI, Body mass index.

### Age- and sex-specific prevalence of cardiometabolic risk factors

**Fig 1:**
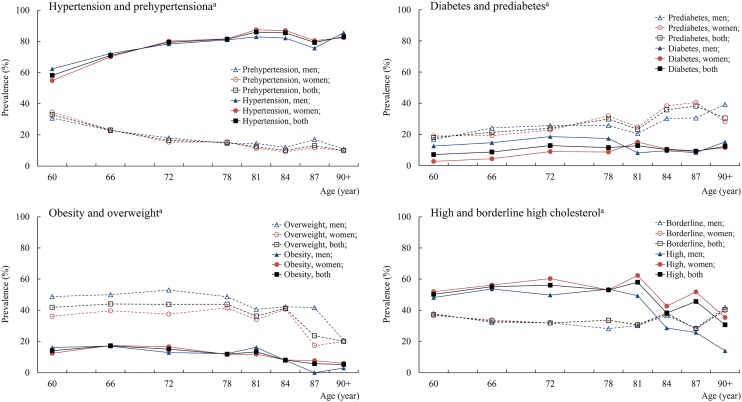
Age- and sex-specific prevalence of cardiometabolic risk factors. ^a^Numbers of subjects with missing value were 48 for prehypertension and hypertension, 244 for prediabetes, 329 for overweight and obesity, and 263 for high and borderline high total cholesterol.

The prevalence of overweight, obesity, and high cholesterol decreased with increasing age (*P* for trend <0.01), whereas the prevalence of hypertension and diabetes increased with age (*P* for trend <0.01) (Fig. 1). In addition, men were more likely than women to be overweight across all age groups (*P*<0.01), and the prevalence of diabetes was higher in men than in women mainly among people aged <80 years (*P*<0.01). There was no sex difference in prevalence of prehypertension, hypertension, obesity, and borderline high cholesterol.

### Aggregation of cardiometabolic risk factors

**Fig 2:**
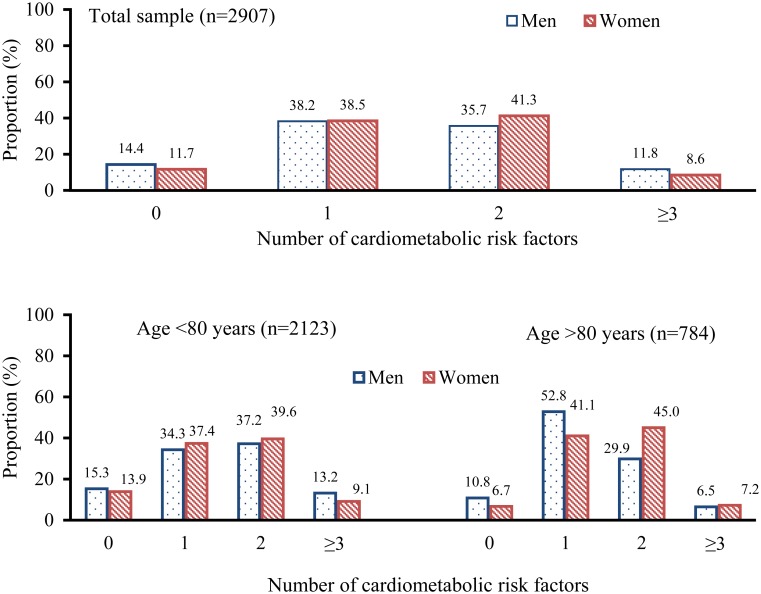
Distribution of clustering of cardiometabolic risk factors by age and sex.

Of the 3363 participants, 456 (13.6%) were excluded due to missing information on blood pressure (n = 48), BMI (n = 329) or total cholesterol (n = 263), leaving 2907 participants for the analysis of cardiometabolic risk factor aggregation. Of the 2907 subjects, 87.3% had at least one cardiometabolic risk factor, 49.0% had two or more, and 9.8% had three or more cardiometabolic risk factors. Women were more likely to have multiple (≥2) cardiometabolic risk factors than men (41.3% vs. 35.7%, *P*<0.01) (Fig. 2). The oldest-old people (>80 years) were more likely to have multiple cardiometabolic risk factors than young-old people (<80 years) (40.6% vs. 38.6%, *P*<0.01).

### Pharmacological treatment of hypertension, diabetes, and high cholesterol, and their control among pharmacologically treated people

Table 3 presents the crude proportions of pharmacological treatment and control of hypertension, diabetes, and high cholesterol. First, 55.5% of people with hypertension were treated with antihypertensive agents; of these, 31.8% achieved the therapeutic goal with blood pressure <140/90 mmHg (controlled I). The rates of pharmacological treatment for hypertension were higher in women than in men (*P*<0.01) and in the oldest-old than in the young-old people (*P*<0.01). The proportion of people who reached the therapeutic goal of blood pressure <140/90 mmHg was higher in the oldest-old than in the young-old people (*P*<0.01). We also estimated the rate of controlled blood pressure according to the newly released JNC-8 guidelines which set the pharmacological treatment goal of blood pressure <150/90 mmHg for adults aged 60 years and above [22] (controlled II). Among the hypertensive patients who were pharmacologically treated with antihypertensive drugs, 49.4% reached the goal of blood pressure <150/90 mmHg, and there was no statistical difference by sex. The proportion was higher in the oldest old than in the young-old people (*P*<0.01).

**Table 3:** Crude proportions of Pharmacological treatment and control of hypertension, diabetes, and high total cholesterol by age groups, sex, and history of cardiovascular diseases.

Pharmacological treatment and control of cardiometabolic factors	All	Sex, n (%)			Age groups (years), n (%)		
	n (%)	Men	Women	*P*	<80	>80	*P*
Hypertension (n = 2496)							
Treatment^a^	1386 (55.5)	448 (51.6)	938 (57.7)	<0.01	761 (48.2)	625 (68.2)	<0.01
Controlled I^b^ (BP <140/90 mmHg)	441 (31.8)	148 (33.0)	293 (31.2)	0.72	197 (25.9)	244 (39.0)	<0.01
Controlled II^b^ (BP <150/90 mmHg)	684 (49.4)	222 (49.6)	462 (49.3)	0.92	337 (44.3)	347 (55.5)	<0.01
Diabetes (n = 318)							
Treatment^a^	160 (50.3)	88 (55.0)	72 (45.6)	0.09	110 (52.6)	50 (45.9)	0.25
Controlled^b^ (HbA1c <7%)	61 (38.1)	38 (43.2)	23 (31.9)	0.15	43 (39.1)	18 (36.0)	0.71
High cholesterol (n = 1523)							
Treatment^a^	381 (25.0)	186 (36.4)	195 (19.3)	<0.01	319 (27.9)	62 (16.4)	<0.01
Controlled^b^ (<6.22 mmol/l)	327 (85.8)	167 (89.8)	160 (82.1)	0.14	275 (86.2)	52 (83.9)	0.58

^a^Treatment referred to individuals with hypertension, diabetes, or high cholesterol who reported taking antihypertensive, cholesterol-lowering, or hypoglycemic medications, respectively.

^b^Controlled groups referred to treated individuals whose blood pressure <140/90 mmHg (controlled I), or <150/90 mmHg (controlled II), HbA1c <7.0%, or total cholesterol <6.22 mmol/l, respectively.

BP, blood pressure; HbA1c, glycated haemoglobin.

Second, of those with diabetes, 50.3% reported using antidiabetic medications, but only 38.1% reached the goal of HbA1c <7%. The proportion of pharmacological treatment for diabetes was higher in men than in women (*P* = 0.09). Of those 158 individuals who did not report using any antidiabetic medications, 68.4% had HbA1c <7%. There was no significant difference in reaching the therapeutic goal by sex or by age groups.

Third, 25.0% of those diagnosed with high cholesterol who reported using cholesterol-lowering agents, and 85.8% of those under pharmacological treatment reached the goal of total cholesterol <6.22 mmol/l. The proportions of pharmacological treatment for high cholesterol were higher in men than in women (*P*<0.01) and in the young-old than in the oldest-old people (*P*<0.01).

### Additional analyses

We performed additional analyses to further investigate whether pharmacological treatment was appropriate among hypertensive patients with blood pressure ≥160/90 mmHg according to the European guidelines [35]. Of patients with blood pressure ≥160/90 mmHg, 69.8% received pharmacological treatment; the proportions of pharmacological treatment were higher in women than in men (72.4% vs. 64.8%, *P*<0.01) and in the oldest-old than in the young-old (83.0% vs. 61.7%, *P*<0.01). Furthermore, the European guidelines recommended the therapeutic goal of blood pressure <140/85 mmHg for patients with diabetes [35]. Overall, 77.4% of hypertensive patients with diabetes reported use of antihypertensive drugs, as compared to the proportion of 52.8% for hypertensive patients without diabetes (*P*<0.01). There was no statistical difference in antihypertensive treatment rates among hypertensive patients with diabetes by sex and age groups. Among patients with diabetes who were treated with antihypertensive drugs, 35.1% reached the goal of blood pressure <140/85 mmHg.

## Discussion

This population-based study of older men and women in central Stockholm, Sweden, showed that hypertension, high cholesterol, and concurrent cardiometabolic risk factors are highly prevalent. Furthermore, suboptimal cardiometabolic conditions such as overweight, prediabetes, and borderline high cholesterol affected up to ~40% of older people. Finally, more than half of patients with hypertension or diabetes and one-fourth of those with high cholesterol reported receiving pharmacological treatment, and by current guidelines less than half of those who were pharmacologically treated for hypertension or diabetes reached the therapeutic goals.

In the WHO supported report, major risk factors for CVDs have been categorized into behavioural risk factors (i.e., tobacco use, physical inactivity, unhealthy diet, and harmful use of alcohol), cardiometabolic risk factors (i.e., hypertension, diabetes, high cholesterol, and obesity), and other risk factors (e.g., age, sex, low education, poverty, inherited disposition, and psychological factors) [6]. In the current study, we focused on prevalence and pharmacological control of major modifiable cardiometabolic risk factors among older adults. The age- and sex-standardized prevalence of hypertension and diabetes in our study population was comparable with other population-based studies of older adults. For example, the MONICA study of people aged 65–75 years in Northern Sweden found that the prevalence was 71.9% for hypertension and 12.0% for diabetes [36]. Similarly, the Rotterdam study of people aged 55 years and older reported a prevalence of 78.6% for hypertension and 10.0% for diabetes [37]. Consistent with previous studies [19,38], we observed an increasing prevalence of hypertension and diabetes with age until around 80 years and over. The U.S. national data have shown that among adults aged ≥65 years, women are more likely than men to have hypertension [19], but we found no sex difference in the prevalence of hypertension. Our data showed that the prevalence of diabetes was higher in men than in women, which is in line with a previous Swedish study [36].

The age- and sex-standardized prevalence of obesity (12.8%) in our study is slightly higher than the report from a recent study of people aged ≥65 years in Stockholm (9.5%) [20]. Furthermore, our study showed a decrease in the prevalence of obesity with increasing age, and there was no sex difference, which are consistent with the previous study [20].

The reference values for defining high cholesterol vary among studies in Sweden [36]. We found that nearly 50% of older people had high cholesterol when the cut-off of ≥6.22 mmol/l was used, which is much higher than reported from the MONICA study of Northern Sweden in 2004 (16.9%) where a cut-off of >7.0 mmol/l was used [36]. Even when the same criteria (>7.0 mmol/l) are applied to our population, the age- and sex-standardized prevalence of high cholesterol (27.4%) was still higher than that from the MONICA study. Data from eight European countries showed a decrease in the mean total cholesterol with age in older adults [31], which is in accordance with our findings. In addition, similar to the previous studies [31,36], we found that women were more likely to have high cholesterol than men, especially among the oldest-old.

People with suboptimal metabolic conditions such as prehypertension, prediabetes, overweight, and borderline high cholesterol are at substantial risk for further progression to hypertension, diabetes, obesity, and high cholesterol, respectively. However, previous research has paid little attention to the prevalence of these suboptimal metabolic conditions among older people. Our study showed that unfavourable cardiometabolic conditions were highly prevalent among Swedish older adults. Notably, we found that prediabetes affected more than 20% of older adults. These findings raise the alarm that people with these conditions should be targeted for early intervention aiming to interfere with progression to cardiometabolic risk factors and reduce risk of CVDs and cognitive dysfunction [39,40].

Very few population-based studies have previously reported the distribution of clustering cardiovascular risk factors among older individuals. A population-based study from Mexico City showed that three-fourths of older adults (age ≥65 years) were exposed to one or more cardiovascular risk factors, including hypertension, diabetes, smoking, hypercholesterolemia, and obesity [41]. Similarly, a population-based study of Chinese older people living in a rural area reported that more than 80% had two or more of the six cardiovascular risk factors (i.e., hypertension, diabetes, high cholesterol, obesity, smoking, and physical inactivity) [21]. Although our study focused on four cardiometabolic risk factors, the results also showed highly prevalent clustering cardiometabolic risk factors in Swedish elderly people: nearly half of older adults had two or more cardiometabolic risk factors and approximately 10% had even three or more cardiometabolic risk factors. The aggregation of cardiometabolic risk factors has been strongly linked not only to cardiovascular events, but also to dementia and mobility limitation among older adults [11,12]. Thus, our study implies that intervention programs targeting multiple cardiometabolic risk factors and unfavourable metabolic conditions among older people living in the communities may help maintain good health in aging for a longer time period.

Previous studies have shown generally insufficient control of hypertension, diabetes, and high cholesterol in older adults [14,33]. Our findings revealed that a considerable proportion of people with hypertension, diabetes, and high cholesterol were not on pharmacological treatment, and elevated blood pressure and high HbA1c in patients with the pharmacological treatments were insufficiently controlled. Compared with U.S. national data of older adults (age ≥65 years) [33], the proportions of both antihypertensive treatment (69.3% vs. 55.5%) and control of hypertension (<140/90 mmHg) (48.8% vs. 31.8%) were lower in the SNAC-K population. According to the newly released JNC-8 guidelines for adults aged 60 years and above [22], up to 49.4% of people with hypertension in SNAC-K population reached the therapeutic goal of blood pressure <150/90 mmHg; the proportion of pharmacologically treated patients that achieved the blood pressure goal was increased by 17.6%, which is similar to the report from the U.S. national survey (19.7%) [42]. In Sweden, people with a blood pressure of 140–159/90–99 mmHg are usually advised to modify their lifestyles before pharmacological antihypertensive therapy was initiated [43]. However, even by the conservative therapeutic goal (blood pressure <160/90 mmHg), the rate of pharmaceutical treatment of patients with hypertension (69.8%) was still insufficient. In addition, our data showed that antihypertensive treatment among patients with diabetes can be improved. Furthermore, we found that women were more likely than men to receive antihypertensive treatment, and the oldest-old people were more likely to reach the goal of blood pressure control than the young-old people. The proportion of antidiabetic treatment in people with diabetes in our study was quite similar to that of the U.S. national data (50.3% vs. 50.9%), but the proportion of those who reached the goal of therapy was lower in our sample (38.1% vs. 50%) [33]. We noticed that among people with diabetes who did not report use of any pharmacological therapy, 68.4% had HbA1c level <7%; these people were likely on a diabetes dietary plan. A previous study reported that the rate of pharmacological treatment among older adults with high cholesterol varies between countries, ranging from ~15% in Germany to ~68% in Mexico [31]. We found a rather low rate of pharmacological treatment for high cholesterol (25%). However, among people who were pharmacologically treated with cholesterol-lowering agents, the rate of reaching the therapeutic goal was comparable to that of a study of Chinese older adults (85.8% vs. 77.2%) [21].

Multimorbidity and chronic conditions are fairly common among older adults [44], which may have an impact on the treatment and control of cardiometabolic risk factors [45]. For instance, a more realistic goal (e.g., HbA1c <8%) for frail older people or older adults with comorbidity or multimorbidity has been proposed owing to the potential risks of tight control of blood glucose may outweigh the benefits [45]. This may partly explain a relatively low proportion of diabetic patients who reached the therapeutic goal of HbA1c in our study. Furthermore, a Danish study of general practice database suggested that blood pressure control rates might also differ substantially within comorbidities, such that blood pressure control was poor among patients with diabetes, whereas presence of CVDs was associated with improved blood pressure control [46].

Strengths of this study include the population-based design and comprehensive assessment of cardiometabolic risk factors. However, this study also has limitations. First, use of medications in our study was based on self-reported information, but previous research has shown that the self-reported use of drugs (e.g., antihypertensive drugs and statins) is relatively accurate compared to pharmacy records [47]. Second, the socioeconomic status of residents in the Kungsholmen district was considered the highest in Sweden. Thus, caution is needed when generalizing our findings to populations in other areas. Finally, data on fasting blood glucose or oral glucose tolerance tests were not available in our study. However, HbA1c has been formerly recommended by the WHO for the diagnosis of diabetes [48].

In conclusion, this population-based study shows that hypertension, high cholesterol, and an aggregation of multiple cardiometabolic risk factors are rather common in older Swedish people. Suboptimal cardiometabolic profiles, such as prediabetes, were also highly prevalent. The pharmacological treatment of people with hypertension, diabetes, and high cholesterol was insufficient, and many people who were under the pharmacological treatment did not reach the therapeutic goals. This study suggests that the cardiometabolic profiles of older adults should be regularly monitored, and that proper management of cardiometabolic risk factors may help improve cardiovascular health of older Swedish people.
